# The net effect: spanning diseases, crossing borders—highlights from the fourth triennial APCA conference and annual HPCA conference for palliative care

**DOI:** 10.3332/ecancer.2013.371

**Published:** 2013-11-05

**Authors:** J Downing, E Namisango, F Kiyange, E Luyirika, L Gwyther, S Enarson, J Kampi, Z Sithole, E Kemigisha-Ssali, M Masclee, I Mukasa

**Affiliations:** 1 Makerere University, Kampala, Uganda; 2 International Children’s Palliative Care Network, Hillcrest 3624, South Africa; 3 African Palliative Care Association UK, London DA15 8AD, UK; 4 African Palliative Care Association, Kampala, Uganda; 5 Hospice and Palliative Care Association of South Africa, Cape Town 7430, South Africa

**Keywords:** palliative care, Africa, policy, integration, consensus development, research

## Abstract

The African Palliative Care Association (APCA) jointly hosted its triennial palliative care conference for Africa with the Hospice and Palliative Care Association of South Africa (HPCA) on 17–20 September 2013 in Johannesburg, South Africa. At the heart of the conference stood a common commitment to see patient care improved across the continent. The theme for the conference, ‘The Net Effect: Spanning Diseases, Crossing Borders’, reflected this joint vision and the drive to remember the ‘net effect’ of our work in palliative care—that is, the ultimate impact of the care that we provide for our patients and their families across the disease and age spectrum and across the borders of African countries. The conference, held in Johannesburg, brought together 471 delegates from 34 countries. The key themes and messages from the conference are encapsulated in ten ‘C’s of commitment to political will and support at the highest levels of governance; engaging national, regional, and international bodies; collaboration; diversity; palliative care for children; planning for human resources and capacity building; palliative care integration at all levels; developing an evidence base for palliative care in Africa; using new technologies; and improved quality of care. Participants found the conference to be a forum that challenged their understanding of the topics presented, as well as enlightening in terms of applying best practice in their own context. Delegates found a renewed commitment and passion for palliative care and related health interventions for children and adults with life-limiting and life-threatening illnesses within the region. This conference highlighted many of the developments in palliative care in the region and served as a unique opportunity to bring people together and serve as a lynchpin for palliative care provision and development in Africa. The delegates were united in the fact that together we can ‘span diseases,’ ‘cross borders,’ and realise the ‘African Dream’ for palliative care.

## Introduction

Palliative care is ‘an approach that improves the quality of life of patients and their families facing the problems associated with life-threatening illness, through the prevention and relief of suffering by means of early identification and impeccable assessment and treatment of pain and other problems, physical, psychosocial and spiritual’ [1]. At the heart of the fourth triennial African Palliative Care Association (APCA) conference and annual Hospice and Palliative Care Association of South Africa (HPCA) conference for palliative care was, therefore, a commitment to improve patient care across the continent.

The theme of the conference, ‘The Net Effect: Spanning Diseases, Crossing Borders’ reflected both this commitment and the drive to remember the ‘net effect’ of our work in palliative care, that is, the ultimate impact of the care that we provide for our patients and their families, across the disease and age spectrum and across the borders of African countries. This theme was visually depicted through the logo featuring the silhouette of a young man and woman standing on a boat and holding a fishing net that covers the globe. This Africa-shaped net reflected APCA’s and HPCA’s joint vision to see palliative care encompassing health systems across Africa, in collaboration with lessons shared from partners around the world, to address the pain—physical, social, emotional, and spiritual—that African patients experience. The young man and woman were symbolic of Africa being the demographically ‘youngest’ continent in the world. Thus, the ripple effect and impact of palliative care policies and services spanning the disease spectrum are likely to have a profound impact on Africa’s next generation and its socio-economic future, as depicted by the fishing boat (with fishing being a symbol of economic livelihood that empowers local communities toward sustainability). The net hangs without lines, representative of the need for palliative care to be inclusive of the linguistic and cultural diversity of patients across Africa, cutting across culture, age and gender, the breadth of the communicable and non-communicable disease (NCD) spectrum, the expanse of life stages, and the need to address the barriers of pain, stigma, and isolation often associated with disease through the World Health Organization’s (WHO’s) physical, psychosocial, and spiritual pillars of palliative care [2].

Two important sessions preceded the scientific programme of the conference: a Ministers of Health Session on Palliative Care and the African Palliative Care Research Network (APCRN) Research workshop.

### Preconference session 1: building consensus among policy makers on the importance of palliative care in Africa

The inaugural African Ministers of Health session on palliative care was attended by 92 delegates from 23 countries (of which 21 were African countries). Of these, 34 delegates represented ministries of health, including four Ministers and Deputy Ministers (from Kenya, Uganda, Malawi, and South Africa) and representatives of Ministers of Health from various countries across Africa. The African Union (AU) Commission was also represented by the Senior Policy Officer of Health, Population, and Nutrition. This session provided an opportunity for Ministers of Health to share experiences and learn from one another in regard to progress being made in palliative care development in their countries based on the WHO-enhanced public health model, focusing on policy development and integration, education, drug availability, and implementation of services. Furthermore, the session provided an opportunity to share the innovative approaches being used to strengthen health systems through the integration of palliative care, as well as the challenges being faced and how these are being addressed to ensure that those in need of palliative care are able to access it. The practical application of the session was reflected in a renewed commitment among Ministry of Health delegations to integrate palliative care into the health systems of African countries through the adoption of the Johannesburg statement titled ‘Consensus Statement for Palliative Care Integration into Health Systems in Africa: Palliative Care for Africa’. This important statement was adopted by all participating Ministers and Deputy Ministers of Health, Ministry of Health delegations from 14 countries, and other key stakeholders, including national palliative care associations and development partners. The statement builds on the AU Ministers of Health Declaration on NCDs, in which African governments committed to the integration of palliative care as well as the AU common position on the use of pain medications, along with other regional and United Nations (UN) declarations. The statement outlines six objectives to guide the integration of palliative care in Africa’s health systems: commitment to (1) the development of policy frameworks that strengthen health systems through the integration of palliative care into hospital and home-based care health services; (2) the integration of palliative care services into national health budgets to ensure sustainable services; (3) the use of the already established global and regional frameworks provided by the AU and WHO; (4) to ensure availability of, and access to, essential medicines and technologies for the treatment of pain and other symptoms, including for children, as well as the procurement and distribution of morphine and to ensure greater availability and access of this main opioid for the management of moderate to severe pain; (5) the integration of palliative care into nursing, medical and other relevant training curricula and preservice training programmes such as those for pharmacists, social workers, psychologists, and the clergy; (6) the sharing of palliative care best practices in clinical care, effective models, and education across the continent and to ensure peer-to-peer learning across borders. These objectives constitute the essential elements of a regional strategy for integrating palliative care into African health systems.

### Preconference session II: strengthening the evidence base for palliative care in Africa

The APCRN session brought together 156 palliative care and health researchers from Africa, Europe, and the United States. The key achievements of the meeting included the development of conceptual notes to conduct research for seven priority research questions, as outlined in the research agenda for palliative care in Africa [3]. The aim of the workshop was to discuss the status of palliative care research in Africa and to share effective strategies for generating and using research evidence in advancing the palliative care agenda on the continent and to continue building capacity for research, in order to sustain a vibrant research culture in Africa. The workshop, chaired by Eve Namisango from APCA, was facilitated by members of the APCRN: Dr Emmanuel Luyirika (APCA), Dr Liz Gwyther (HPCA), Prof. Lukas Radbruch (University of Bonn), Assoc. Prof. Richard Harding (King’s College London), Prof. Julia Downing (Makerere University), Dr Liz Grant and Prof. Scott Murray (University of Edinburgh). Eve Namisango, the Research Manager at APCA, noted the importance of research for patient and family outcomes and emphasised the need to sensitise ethical review boards to the vital importance of palliative care research. Participants were divided into groups to identify ways to collaborate and engage in the research agenda. The priority areas discussed included the palliative care needs of children, the impact of palliative care training on care and practice, palliative care integration, outcomes of care, palliative care needs assessment, assessment of drug availability and accessibility, and volunteers in palliative care. Throughout both the workshop and the main conference, the commitment and enthusiasm to developing a palliative care evidence base through research were evident, with Prof. Murray encouraging delegates to commit to writing and publishing their research.

## Conference background

Since the Cape Town Declaration in 2002 [4], there have been rapid advances in the development of palliative care in Africa [5], with the commitment to pain relief being a human right and palliative care being integrated into national health policies/strategies. This conference provided a platform for sharing and demonstrating significant advances in the provision of palliative care in the region. While there is still a long way to go—at least 28 countries in the region still have little to no access to palliative care [6], and at least 43 countries in the region have little to no access to children’s palliative care [7]—there was a sense of having moved in the right direction. Through collaboration and advocacy, strides have been made to advance palliative care in Africa, not only in terms of service provision but also in terms of quality of care and the quality of services that are provided to patients. Various presenters emphasised the potential of palliative care in transforming health systems in Africa due to its family-centred and multidisciplinary approach. Presenters also noted the key role of palliative care in areas of global health, including the prevention and control of both communicable diseases and NCDs. Malik Jaffer of IntraHealth reflected on progress over the past three years since the previous conference, stating, ‘Palliative care in Africa has been elevated in the last three years. We are engaging at such a different level, practically and conceptually. At the last conference (2010), the talk was all about how to do service delivery better; now we are focusing on how to integrate palliative care into health systems, into social systems, and we are engaging Ministers of Health. We are talking about how to change the way that palliative care is conceived and the way it engages global health’. [8]

## Conference summary

The conference, held at the Birchwood Convention Centre in Johannesburg, brought together 471 delegates from 34 countries from around the world. Of these, 24 were African countries, with delegates from Botswana, Burundi, Cameroon, Cote D’Ivoire, Democratic Republic of Congo (DRC), Ethiopia, the Gambia, Ghana, Kenya, Malawi, Morocco, Mozambique, Namibia, Nigeria, Rwanda, South Africa, Sudan, Swaziland, Tanzania, Togo, Uganda, Zambia, Sierra Leone, and Zimbabwe. Other countries represented included Canada, England, Italy, Hungary, Germany, Malaysia, Scotland, Sweden, and the United States. The conference brought together the continent’s clinicians, academics, human rights advocates, lawyers, clergy, researchers, social workers, policy makers, Ministry of Health officials, donors, members of the press, and national palliative care associations to share lessons and adopt best practice for strengthening and integrating palliative care. Partner agencies and governments were represented by decision makers from within and beyond Africa, along with technical experts in fields such as advocacy, research, organisational development, human rights, communications, and fundraising. The conference was organised into four tracks: (1) spanning diseases with palliative care, (2) crossing borders with health systems strengthening, (3) showcasing impact, and (4) sustaining our work. The scientific programme included a variety of plenary sessions (11 papers), 64 oral breakout presentations, 16 workshops, and 104 poster presentations. Oral and poster abstracts were accepted from 27 countries, 21 of them African with institutional representation spanning organisations that have spearheaded the development of palliative care in the region, alongside institutions that are just beginning to integrate palliative care, thus demonstrating the breadth of delegates represented.

During the conference, lifetime achievement awards were presented to individuals who have made a significant impact in contributing to palliative care development on the continent. The two lifetime achievement awards went to Prof. Anne Merriman of Hospice Africa Uganda (HAU) and Kath Defilippi (formerly HPCA), both founding board members of APCA. Kath Defilippi was also awarded the ‘best research abstract’ presented at the conference. A pioneering leadership award was presented to Dr Faith Mwangi-Powell, APCA’s founding Executive Director. Additional recognitions included Edith Akankwasa (Mildmay Uganda) for best poster presentation and Assoc. Prof. Richard Harding (King’s College London) for his commitment to research within the region. With funding from the Open Society Foundations, a series of public health policy, advocacy, and journalist awards were presented, aimed at recognising excellence in advancing palliative care on the continent and excellent press coverage of issues pertaining to palliative care in Africa, respectively. The Kenya Ministry of Health was awarded the Public Health Policy Award for its commitment to championing access to palliative care, HAU was awarded the institutional advocacy award for its commitment to palliative care provision in Uganda and across Africa, and Dr Zipporah Ali of Kenya Hospices and Palliative Care Association (KEHPCA) was awarded the individual advocacy award for her commitment to palliative care provision in Kenya. Madeleine French from the United Kingdom was awarded first place in the Palliative Care Journalist Award, for her article ‘Opioids for the Masses’, published by *Think Africa Press*. Second place was awarded to Vennessa Scholtz from South Africa for her article ‘A Caring Approach’, published in the Bellville/Durbanville *Tygertalk*, and third place was awarded to Mallick Mnela from Malawi for his article ‘Of Malawi’s Painfully Slow Palliative Care Roll-Out’, published on the website Zodiak Online. Wilma Stassen of South Africa was awarded fourth place for her article ‘Destined to Live and Die in Pain’, published on Health-e.

### Key conference themes

The key themes and areas of commitment from the conference can be summed up in the following ten ‘C’s:

1. Commitment and political will to integrate palliative care into national health systems
2. Commitment to engage national, regional, and international bodies
3. Commitment to diversity
4. Commitment to collaboration and working together
5. Commitment to palliative care for children
6. Commitment to plan for human resources and capacity building in palliative care
7. Commitment to the integration of palliative care across health systems and the disease spectrum as well as into services of special needs populations
8. Commitment to develop an evidence base
9. Commitment to use new and emergent technologies to advance palliative care on the continent
10. Commitment to improved quality of care

**1. Commitment and political will to integrate palliative care into national health systems**

For the first time, high-level African Ministry of Health representatives convened to address palliative care integration in Africa. A pre-conference Ministers’ of Health session on palliative care was hosted by South Africa’s Deputy Minister of Health, the Hon. Dr Gwen Malegwale Ramokgopa (see above). This meeting was an outcome of years of advocacy for palliative care across the region, and efforts to get palliative care recognised as a human right for all children and adults with life-limiting and life-threatening illnesses and therefore integrated into Africa’s health systems. The meeting was a strategic turning point in the effort to reduce suffering among patients with life-limiting illnesses and served as a significant step forward in moving toward integrating palliative care into health systems throughout the region.

Throughout the meeting, speakers from various ministries of health, including the participating ministers of health, emphasised their commitment to ensuring that palliative care is put on the health agenda of their countries and that services be integrated and provided to the general population within their countries. The Hon. Sarah Achieng Opendi from Uganda noted that the need for palliative care cannot be underestimated and while Uganda has moved far in the provision of palliative care, it is still only accessible to 10% of those who need it. Later in the conference, she challenged the Ugandan delegation as to whether they are ready and able to meet the demand if she committed to palliative care advocacy and engagement at the highest levels of government (the Cabinet and Parliament). The Hon. James Macharia, Cabinet Secretary of the Ministry of Health, Kenya, also stated. ‘I am glad that the issue of palliative care has been given prominence because we have taken it for granted’.

Commitment and political will were also demonstrated throughout the conference. Many government representatives stayed for the entirety of the conference, actively taking part in sessions and workshops and affirming their commitment to palliative care development in their respective countries. One government official commented that ‘The conference has been challenging—as a government person we have to take on palliative care’; another said ‘I am not sure how we have been managing not to provide palliative care for so long’. This commitment from governments, along with the political will to move the agenda for access to palliative care forward, remains an essential component of the WHO public health model for the development of palliative care [9]. Her Royal Highness Princess Dina Mired, Director General of the King Hussein Cancer Foundation in Jordan opened the first plenary session by stating in a video address to delegates: ‘Governments are primarily focused on drug control and addiction. But one issue should not cancel out the other… Palliative care has slipped through the gaps. When you see a loved one grimacing with pain day and night, you understand palliative care is not a choice. It’s a human right’.

Political will and commitment were also highlighted as essential ingredients to the development and integration of palliative care into national legislation, policies, strategies, and plans. This was evident when presenters from Rwanda (Dr Christian Ntizimira, Kibagabaga District Hospital), Swaziland (Ntombi Ginindza, Ministry of Health), Mozambique (Lidia Monjane, Mozambique Palliative Care Association), The DRC (Dr Paul Pilipili-Hangi, Directorate of Primary Health Care and Public Health), Uganda (Rose Kiwanuka, Palliative Care Association of Uganda), and Zimbabwe (Eunice Granganga, Hospice Palliative Care Association of Zimbabwe) shared their experiences on palliative care policy development at a workshop led by Mary Callaway of the Open Society Foundations and Fatia Kiyange of APCA.

**2. Commitment to engage national, regional, and international bodies**

‘Crossing borders’ was part of the theme of the conference as opportunities for collaboration were a key part of many of the presentations. Throughout the conference, speakers emphasised that palliative care is a human rights issue; thus there is a need to ensure that it continues to be among national, regional, and global health priorities. The need to engage international and regional bodies such as the UN; the AU Commission; the WHO’s Regional Office for Africa; the East African Community; the Southern African Development Community; and the Economic Community of West African States was seen to be critical in achieving universal access to palliative care. Throughout the conference, the issue of access and availability of opioid medications was raised. While political will and commitment are key to following through on this commitment for each individual country, this was also an area where working closely with national, regional, and international bodies, such as the national palliative care associations, the regional associations, and organisations such as the Pain and Policies Studies Group, the Global Access to Pain Relief Initiative (GAPRI), the Worldwide Palliative Care Alliance (WPCA), the International Hospice and Palliative Care Association (IAHPC), and the ICPCN, to name but a few. As representatives of many of these organisations were present at the conference, alongside presentations addressing key issues, delegates had the opportunity to collaborate on initiatives to move the palliative care agenda forward within their respective institutional mandates. As one delegate commented, ‘We need to collaborate, not just with each other, but also with those outside of palliative care in order to improve care’. This comment reflects a commitment to strengthen existing and developing new partnerships with a range of national, regional, and international bodies.

**3. Commitment to collaborate**

While there were no specific presentations on the need to work together, collaboration was evident throughout the conference, with delegates sharing examples of best practice and lessons learnt and exchanging contact information. One of the objectives behind APCA’s establishment in 2004 was to bring together individuals working in palliative care across the region to work together for the further development of palliative care. In many ways, APCA has been pursuing this role, and by cohosting this conference with HPCA, APCA has demonstrated this commitment. When asked about the conference, delegates commented positively on the atmosphere, the drive toward collaboration, and the way that individuals and organisations are coming together to develop and promote palliative care services across the region. Speaking to the delegates from Uganda at a side meeting, the Minister of State for Primary Health Care in Uganda, advised the palliative care technical team from her country to meet upon return from the conference to discuss next steps for the palliative care agenda and then brief her on how the agenda can be moved forward with her support. She noted that the door to her office is open, demonstrating the commitment at the highest level to work together.

**4. Commitment to diversity**

The definition of diversity has been cited as a concept that ‘encompasses acceptance and respect. It means understanding that each individual is unique, and recognising our individual differences’ [10]. Throughout the conference, there was an understanding that we are all unique, that we have our own strengths and weaknesses, but that we need to come together in palliative care through mutual acceptance and respect. This conference showcased diversity, both in terms of diversity within teamwork and also within palliative care provision. Plenary speakers represented a range of professions, countries, and backgrounds, including palliative care doctors (Dr Liz Gwyther, Dr Emmanuel Luyirika), public health experts (Dr Allison Russell), the mother of a cancer survivor and Director General of the King Hussein Cancer Foundation (HRH Princess Dina Mired), technical advisor and expert in health systems strengthening (Malik Jaffer), a surgeon and expert in health service planning and development (Dr Isaac Ezaati), a social worker, social sector planner, and expert in programming for palliative care (Fatia Kiyange), an HIV physician and expert in health economics and one of the architects of PEPFAR (Prof. Joseph O’Neil), an organisational development expert (Andre Wagner), and a nurse and international advocate for children’s palliative care (Joan Marston). There was a commitment to broadening the professional base of palliative care teams, including wider professional input such as from medical anthropologists, lawyers, and the media. Likewise, when addressing the palliative care needs of individuals, there were presentations about providing care for a broader range of individuals such as the deaf (Francesca Tong, HPCA), older persons (Esther Kavuma, Mildmay Uganda), those in prison (Zodwa Sithole, HPCA; Gavan O’Sullivan, South Africa), children and young people (Dr Cornelia Drenth, South Africa; Prof. Julia Downing, Uganda; Stephen Connor, United States; Chenjerau Sisimayi, Zimbabawe; Busi Nkozi, South Africa). There was also a session addressing the provision of palliative care in Portuguese and French speaking countries in Africa (Lidia Monjane, Mozambique and Dr Paul Pilipili-Hangi, DRC). A strong focus on the needs of patients and their families was seen through a session on the ‘caregiver’s journey’ presented by Heleen Van Huyssteen from South Africa, along with the plight and needs of professional care givers in palliative care, such as nurses. In summary to the value of the conference’s representation of diversity, one delegate noted that ‘Palliative care is for all and we have been able to expand our services—it shows that we are looking out and growing up’.

**5. Commitment to palliative care for children**

Children are not ‘just little adults’; they need palliative care specific to their needs and ages. In her presentation on children’s palliative care in the region, Joan Marston of the International Children’s Palliative Care Network (ICPCN) noted that while ‘Africa has the greatest gaps, we also have some tremendous programmes, and real progress has been made over the past few years’. While in 2007, at the second triennial APCA conference in Kenya, there had been a feeling that children were being ignored; this was definitely not the case at this conference, with many papers being presented on children’s palliative care. The papers addressed progress, challenges, lessons, and recommendations for the future of children’s palliative care (Dr Alison Russell, USAID); paediatric palliative care assessment—the HPCA CARES Score (Dr Corenlia Drenth, HPCA); development of an e-learning programme for children’s palliative care (Prof. Julia Downing, ICPCN); mobilising a network to stop children suffering (Joan Marston, ICPCN); and advocacy for children’s palliative care (Busi Nkosi, ICPCN). It was great to hear about some of the research that is ongoing within the field of children’s palliative care, notably a three-country assessment (Kenya, Zimbabwe, and South Africa) of the need for palliative care for children (Stephen Connor and Chenjari Sisimayi, ICPCN). Workshops also offered an important opportunity for delegates to discuss issues around children’s palliative care, particularly within the workshops on research, children’s palliative care, the use of the APCA Africa Palliative Care Outcome Scale (APCA POS), issues around legal and human rights, and integrating palliative care, to name a few. Reflecting on the theme of this year’s World Hospice and Palliative Care day on 13 October, Joan Marston (ICPCN) also reminded delegates of the need to dispel myths around palliative care for children, the need to increase our knowledge, the need to be active in reaching children in pain and most importantly the need to listen to children, hear their needs, and provide care for them now [11].

**6. Commitment to plan for human resources and capacity building in palliative care**

The availability of human resources for palliative care within the region has always been a challenge, with palliative care not being perceived as essential and not yet recognised as a discipline in most countries in sub-Saharan Africa. These perceptions pose a challenge for the allocation of staff, along with the lack of a career pathway for those involved in palliative care. Even so, finding a skilled workforce that can deliver care and making sure they can be trained, supported, and sustained is crucial if we are to meet the needs for palliative care within the region. At the Ministers’ Session, participating Ministers of Health highlighted the challenge of not having a skilled workforce for the provision of palliative care, noting the need for specialised training for local health care providers and suggesting measures to retain trained staff in such specialised segments of health such as palliative care. Malik Jaffer (IntraHealth) explored some of the issues of human resource planning and shared a number of tools used in other aspects of health care. He encouraged participants to utilise available tools and resources to help organisations and national palliative care associations to engage with their health and social services to strengthen planning for human resources in palliative care. Andre Wagner (HPCA) addressed the issue of human resources further in terms of ensuring and enabling effective leadership and management within palliative care programmes. As the palliative care environment has been and will continue to be a rapidly changing environment, we need to be prepared to adapt and develop our programmes to meet the needs of the individual and their family members. Capacity building is essential for palliative care provision to continue to develop and for quality care to be provided. Throughout the conference, there was an emphasis on education, addressing the issues of integration into medical and nursing curricula (Dr Zipporah Ali, KEHPCA); training pharmacists (Dr Esther Muinga, Kenya); developing e-learning programmes (Prof. Julia Downing, ICPCN), and monitoring the impact of education and training (Irene Kambonesa, APCA). Examples of best practice were shared from different countries and lessons learnt were identified, including considerations when integrating palliative care into pre- and in-service health education and recommendations for how to take a human resource planning and capacity building agenda forward in the region.

**7. Commitment to the integration of palliative care across health systems and the disease spectrum**

The issue of integration of palliative care into existing structures and programmes is key to the ongoing sustainability of services and in increasing access to all who need its services. Integration was addressed in a variety of ways, whether through the integration of palliative care into education programmes (Dr Zipproah Ali, KEHPCA); integrating palliative care into hospitals (Dr Liz Grant, University of Edinburgh); integrating palliative care into health systems (Dr Asaph Kinyanjui, KEHPCA; Eunice Garanganga, HOSPAZ); integrating palliative care into special needs populations such as prisoners (Zodwa Sithole and Graeme Wilkinson, HPCA), or integrating outcome mapping into monitoring and evaluation (M&E) systems (Chenjerai Sisimayi, Zimbabwe). With the enormous need for palliative care in the region, and the reality of limited human and financial resources, integration is key to the ongoing development of palliative care in the region, and delegates were committed to finding new and innovative ways of doing this. The need to integrate palliative care in the services provided by other disciplines was also recognised, for example, the integration of legal and human rights issues, and the role of legal practitioners was reviewed at a workshop led by Dr Liz Gwyther of HPCA and Emmanuel Kamonyo of the Open Society Initiative for Southern Africa. Dr Isaac Ezaati, a senior surgeon in Uganda and Director of Health Services Planning and Development, shared his experience as a surgeon and reiterated the need to ensure that palliative care is well integrated in surgery with appropriate guidelines developed for surgeons. Dr Abraham Endeshaw Mengistu, the Director of Medical Services and representative of Dr Kebede Worku, the State Minister of Health of the Federal Democratic Republic of Ethiopia’s Ministry of Health, noted of this session: ‘The plenary session focusing on the experiences of a surgeon from Uganda was very inspiring as professionals normally give up in terminal illness and stop caring. He convinced us that there is a lot that can still be done to make a patient comfortable and improve their quality of life. He also convinced us that there is an important role for surgery in palliative care’. Irrespective of the disease or the point of contact for the patient, speakers highlighted the importance of the provision of palliative care starting from the point of diagnosis as an important aspect of palliative care integration in Africa.

**8. Commitment to develop an evidence base**

Over the years, there have been numerous reports citing the lack of an evidence base for palliative care within the region, both for adults and for children [12–14], along with a reported need among care providers and advocates for robust research in the region. Even so, an evidence base to make the case for palliative care in Africa has in many ways remained inadequate [5]. Throughout the Ministers’ Session, there was a call from governments for an increased evidence base, necessary for advocates to call for the adoption of national, regional and global indicators for palliative care provision, and showcase the quality and cost-effectiveness of palliative care services. In her presentation on indicators for palliative care, Emilly Kemigisha-Ssali, APCA’s Senior M&E Officer, shared APCA’s plans for tracking the progress of palliative care interventions and the development of regional indicators for palliative care. Other presentations, such as those by Prof. Lukas Radbruch and Prof. Julia Downing, addressed research priorities for palliative care in general and children’s palliative care specifically, identified through consensus building approaches. A number of presenters reported the findings of more focused research studies such as the prevalence and burden of physical and psychological symptoms in patients with advanced heart failure (Dr Richard Harding); evaluating the palliative care needs of hospitalised tuberculosis patients (Kath Defilippi); factors influencing morphine use in hospitals in Zambia (Elisabeth Mushinda); a survey of opioid prescribing habits in Kenyatta National Hospital (Dr David Wata); prevalence of NCDs among HIV clients accessing care at Mildmay Uganda (Esther Kavuma); and the impact of a palliative care link-nurse programme at Mulago Hospital, Kampala (Mwazi Batuli), among others.

**9. Commitment to use new technologies**

As the cost to attend international palliative care conferences is largely beyond the means of many African delegates, special effort was invested to ensure key messages from the conference were disseminated via social media channels. For the first time, African and international partners could follow progress at the APCA conference through media coverage and through social media. Delegates were encouraged to tweet using the hashtag #hpmafrica, where commentary on various sessions were posted and made visible through Visibletweets and up-to-date conference developments disseminated broadly. Targeted session summaries were published each day on the global news platform for hospice and palliative care news, ehospice (https://www.ehospice.com), and an article ‘The Need to Rethink Palliative Care in South Africa’ [15] was published by the Health-e South African health news service. A special press conference was held for Ministers of Health to address the media following the Ministers’ Session. The investment in a media and communications team was therefore integral to disseminating coverage of the conference.

Alongside the use of social media to generate discussion, papers were presented and workshops held to discuss the role of emergent technologies in palliative care. A workshop held on the first day addressed the issue of engaging the media and using innovative technology such as e-learning, ehospice, and mobile phones (David Praill, WPCA and Kate Jackson, ehospice). Delegates at this workshop were encouraged to think about different stories to write about and were given tips on how to do so. Maeghan Orton from Medic Mobile explored innovative ways of using mobile phone technology for health care, providing examples of innovative mHealth programmes currently underway and encouraging delegates to think about how such technology could be leveraged for palliative care. Opportunities for e-learning were also discussed along with lessons learnt by the ICPCN in developing their programmes. Shelley Enarson (APCA) presented storytelling techniques to showcase the impact of palliative care on patient outcomes and also presented on increasing media coverage in palliative care as essential to raising awareness around what palliative care is and why it matters. During the workshop ‘Marketing Palliative Care: What Has Worked and What Has Not Worked’, Malik Jaffer (IntraHealth) hosted a talk show simulation with panelists that included specialists in oncology, branding, social media, and two award winning journalists. The talk show audience was encouraged to think about the best ways to engage the media to raise awareness among policy makers and the general public around the importance of palliative care service provision in Africa (Malik Jaffer, IntraHealth; Shelley Enarson, APCA).

**10. Commitment to quality**

Quality in palliative care is about providing holistic care to those who need it, including physical, psychological, social, and spiritual care. Throughout the conference, these dimensions of care were addressed in various ways, including the use of cancer pain management guidelines (Dr Liz Gwyther, HPCA); the use of alternative medicines (Dr Israel Kolawole, Nigeria); addressing the caregiver’s journey (Heleen Van Huyssteen, South Africa); spirituality in palliative care (Fr Richard Baeur, Namibia; Prof. Anne Merriman, Uganda); and wound management (Prof. Lukas Radbruch, Germany).

## Conclusion

The last few years have seen significant developments in palliative care within Africa. This fourth triennial APCA conference and annual HPCA conference for palliative care highlighted many of these developments and served as a unique opportunity to bring delegates from multidisciplinary backgrounds together and act as a lynchpin for palliative care provision and development in Africa. The conference inspired policy makers, donors, and international health care leaders, instilling greater confidence and motivation among Africa’s palliative care community, and renewing the belief that our dedication and hard work will achieve the kind of palliative care that we ourselves would all want to receive, for all those who need it within the region. By crossing borders between disciplines and sharing best practices from around the world, we can ultimately achieve the aim of making palliative care a human right for all [2]. Delegates were united in the reality that together we can ‘span diseases’, ‘cross borders’, and realise the ‘African Dream’ for palliative care.

## Conflict of Interest

The authors declare that they have no conflict of interest.
